# Editorial: Bioactive compounds, functional ingredients, antioxidants, and health benefits of edible plants, volume II

**DOI:** 10.3389/fpls.2025.1666398

**Published:** 2025-08-06

**Authors:** Eman A. Mahmoud, Hosam O. Elansary

**Affiliations:** ^1^ Department of Food Science, Faculty of Agriculture, Damietta University, Damietta, Egypt; ^2^ Department of Plant Production, College of Food & Agricultural Sciences, King Saud University, Riyadh, Saudi Arabia

**Keywords:** bioactive compounds, natural products, medicinal plants, antioxidants, pharmaceutical

Bioactive substances with physiological effects, including antibacterial, anti-inflammatory, antioxidant, and anticancer properties, are abundant in edible plants (Yousaf et al., 2024). In order to maintain the quality and safety of both fresh and processed foods, natural plant extracts are widely employed to extend their shelf life. In the food and human nutrition industries, phytochemical analyses of extracts and biological activities of different plant parts are also crucial. By creating new uses for the food industry, they could open the door for the commercialization of additional plants. A potential area of study for plant breeders, producers, and food processors is plant bioactive chemicals (Mazhar et al., 2025). The agriculture and food sectors around the world are very interested in developing new technical methods for the production and processing of edible plants. The goal of the producer and related food businesses is to increase the production of secondary metabolites (like polyphenols) and create new functions. The generation of particular secondary metabolites with significant uses is closely linked to the use of bioreactors in tissue cultures.

In the Research Topic, the work of Kumar et al. revealed that the Thiol-based redox sensing of 16 diverse genotypes of wheat grains affects various metabolic pathways. They found correlation between the accumulation of macro-/micronutrients such as iron and zinc inside the grains and the thiol and disulfide content. They proved a negative relationship between thiol content and nutrient-linked traits such as total protein, gluten, and phytic acid. In a related study, Xu et al. examined the metabolomic profiles and potential health benefits of Euchresta japonica tissues. They found 2,140 metabolites, including phenolic acids, flavonoids, alkaloids, lipids, and amino acids, which were concentrated in the plants’ roots. The majority of these molecules are linked to active medicinal components that are resistant to nine human diseases.

Hou et al. used UPLC-MS/MS to identify 1929 distinct pharmacological compounds *in Dendrobium officinale* stems cultivated in various conditions. When compared to other habitats, they discovered that the stone epiphytic environment had higher levels of 58 primary and secondary metabolites. They came to the conclusion that growing environment affects the amount and composition of metabolites in *D. officinale* stems. In the same context, the chemical and bioactive characteristics of Chinese peony flowers were investigated by Peng et al. Out of the 150 chemical components found in the study, over 50 were reported for the first time from this species, and there were also possible novel substances found. Four cultivars and flower sections were clearly distinguished by 67 quantified or semi-quantified targeted metabolomics analyses. CPF showed strong antioxidant properties and anti-inflammatory actions by lowering the release of nitric oxide, IL-6, and TNF-a in LPS-induced macrophages. They concluded the potential of these flowers to promote health by finding a link between total phenolic content and DPPH ABTS and FRAP antioxidant capabilities.

The effects of methyl jasmonate (MeJA) on blueberry ripening fruits were examined by Vasques-Rojas et al., with a focus on the antioxidant enzyme activity (APX, CAT, SOD, and POD), nutritional qualities, and cell wall composition. They discovered that MeJA increased the antioxidant enzymes’ activities and that anthocyanin accumulation maximized in the blue stage. They came to the conclusion that MeJA reduces fruit softening during storage and preserves cell wall integrity. In the same context, Coban et al. investigated the bioactive substances in 31 genotypes of fenugreek (*Trigonella foenum-graecum*) cultivated in various conditions. They discovered that genotype, environment, and their combination had a substantial impact on the levels of diosgenin, trigonelline, and 4-hydroxyisoleucine measured under irrigated and non-irrigated conditions.

Amangeldinova et al. used the LC-MS/MS technology to quantitatively identify 53 phytochemicals in the roots of *Rheum tataricum* L. Cyranoside, epicatechin, catechin, and chlorogenic acid were all abundant in the extracts. Stronger antioxidant qualities were shown by extracts made using ultrasonic extraction. The UAE-M-4h extract has the highest total phenolic concentration (213.44 mg GAE/mL). For both Gram-positive and Gram-negative bacteria, the extracts made with UAE-MeOH-2h-4h, UAE-EtOH-2h-4h, Sbc-EtOH-E-140-60-80, Sc-90 atm, and Sc-400 atm exhibited antibacterial activity at different rates (MIC range: 31.25 to 250 μg/mL).

Cheng et al. used UPLC-QTOF-MS/MS to examine the seasonal variation and quality variations of flavonoids between *Scutellaria barbata* roots and aerial parts. They found 46 chemicals, mostly flavonoids, in *S. barbata*. They discovered that the root accumulates flavonoids lacking this 4′-hydroxyl group in a manner that is dependent on the season, but the aerial parts collect flavonoids with this group. While roots accumulated the majority of flavonoids in the fall, aerial portions accumulated the most in the spring. Viteri et al. investigated *Wigandia ecuadorensis* antimicrobial, enzymatic inhibitory, antioxidant, and phytochemical profile properties. With the ethyl acetate fraction being the most active, they discovered that the methanolic extract and its subfractions demonstrated a notable antioxidant activity. Its high total phenolic content (357.47 mg GAE/g) was associated with this antioxidant activities. Their Minimum Bactericidal Concentration (MBC) values for *S. aureus, E. faecalis*, and *E. coli* ranged from 1.56 to 6.25 mg/mL, indicating strong antibacterial activity against human pathogen strains. Forty chemicals, including phenolic acids, flavonoids, saponins, terpenes, and fatty acyls, were found using UHPLC-QTOF-MS analysis.

Gharzouli et al. studied Antifungal effect of Algerian essential oil of *Cymbopogon citratus* and *Citrus limon* nanoemulsions to control the fungal infections by *Penicillium digitatum* and *Penicillium expansum* in Thomson Navel oranges (*Citrus sinensis* L. Osbeck). They found that applying nanoemulsified *C. limon* and *C. citratus* as a coating on orange fruits reduced the spread of both fungi compared to the control and reduced the negative changes in quality parameters during storage, such as weight loss, firmness, TSS, TA, pH, and ascorbic acid content.

Jain et al. reviewed *Amorphophallus konjac* traditional uses, bioactive potential, and emerging health applications. They found remarkable health benefits, including improving metabolic health through weight management, blood glucose stabilization, and lipid profile enhancement. Additionally, they have anti-inflammatory, immune-regulatory, and gut-healthy properties. They can be used to treat colorectal cancer (CRC), hyperthyroidism, and inflammatory bowel disease. They also showed difficulties in preserving purity and molecular uniformity, as well as possible adverse effects such gastrointestinal distress and allergenicity.
